# E-Learning Success Model in the Context of COVID-19 Pandemic in Higher Educational Institutions

**DOI:** 10.3390/ijerph19052865

**Published:** 2022-03-01

**Authors:** Fakher Jaoua, Hussein M. Almurad, Ibrahim A. Elshaer, Elsayed S. Mohamed

**Affiliations:** 1 Department of Business Administration, College of Economics and Administrative Sciences, Imam Mohammad Ibn Saud Islamic University (IMSIU), Riyadh 11432, Saudi Arabia; hmalmurad@imamu.edu.sa (H.M.A.); esmohammed@imamu.edu.sa (E.S.M.); 2 Department of Management, College of Business Administration, King Faisal University, Al-Ahsa 31982, Saudi Arabia; 3 Tourism Studies Department, Faculty of Tourism and Hotels, Suez Canal University, Ismailia 41522, Egypt; 4 Faculty of Commerce, Tanta University, Tanta 31521, Egypt

**Keywords:** e-learning success model, e-learning effectiveness, e-learning system, e-learning readiness, interactivity, resistance to change, COVID-19 pandemic, higher educational institutions, Imam Mohammad Ibn Saud Islamic University

## Abstract

Nowadays, the extensive use of e-learning in higher educational institutions in many countries leads us to apprehend the reality, precisely the key success/failure factors of the implementation, of e-learning systems in these institutions. This motivation becomes more and more important, inevitable, and urgent, especially for institutions that have heavily adopted e-learning systems under exceptional conditions without any prior planning, such as the COVID-19 pandemic. From this perspective, this research aimed to provide an e-learning success model in the context of the COVID-19 pandemic by assessing e-learning effectiveness and by investigating the key antecedents of e-learning effectiveness. The literature review led to the identification of four main factors influencing e-learning effectiveness: The e-learning system, e-learning readiness, interactivity, and resistance to change. These four variables constituted the antecedents of an effective e-learning system, which was tested in a KSA context. A structured survey, including a sample of 1202 students from Imam Mohammad Ibn Saud Islamic University was used to examine the linkages among our proposed model. The model, with a total of ten direct and six indirect relationships, was tested by using structural equation modeling. The research findings indicate that effective e-learning is supported by the interactions between four factors: the e-learning system, e-learning readiness, interactivity, and resistance to change.

## 1. Introduction

In recent years, the world has seen an essential development in higher educational institutions (HEI) with the rapid growth of information and communication technologies (ICT) and of computer software [1]. This growth has created an unprecedented revolution in learning or teaching strategies, precisely in distance education. New concepts have received attention, such as e-learning (EL) or online learning, and blended learning (or hybrid learning), which combines face-to-face learning and EL. Hence, EL has become extensively used in HEI and has, for several years, been seen as one of the most important systems for education improvement in some countries. For example, according to [2], in the USA in 2000–2001, 90% of public HEI for the short cycle and 89% of public HEI for the long cycle offered distance education, with enrolments of 47.84% (1,472,000) and 30.71% (945,000), respectively, out of a total enrolment of 3,077,000. In the UK, EL has been adopted by 95% of all HEI [3]. The budget for EL in India increased from $2 billion to $5.7 billion between 2016 and 2020 [4].

This movement from traditional learning towards the EL has shown a growing interest focused on a wave of comparative studies focused on the differences between distance education and traditional education [5], student learning results [6], the quality of EL [7,8], and the weaknesses and strengths of EL [9]. This proliferation of research has contributed to the development of several models of e-learning system success (ELSS).

Despite this focus on e-learning systems (ELS), many educational institutions have failed in the implementation of ELS because they have not considered this important issue of ELS assessment [7]. For example, the UK Government spent $113 million in 2000 to establish an EL project called United Kingdom e-University (UKeU). However, the government announced, in 2004, that the UKeU had failed. This failure was related to the failure to meet recruiting targets [8] and to students’ refusal of online materials and preference for traditional texts as a support for their education [10]. The same results were found in Austria, where students still preferred traditional learning based on interpersonal relations [11]. Online New York University is another example of failure for economic reasons [8]. Thus, facing this problem of failure, the ELS needs more investigation, principally on the assessment of the success and/or their effectiveness [12].

Of course, these previous issues should be studied in depth because of their unknown effects in educational institutions, especially for institutions that had adopted ELS under exceptional conditions, without any prior planning, such as the COVID-19 pandemic. This is the case for many universities around the world and of Imam Mohammad Ibn Saud Islamic University (IMSIU) in the context of the COVID-19 pandemic. In fact, following the sudden emergence of the COVID-19 pandemic and its rapid contagiousness around the world in March 2020, an unprecedented context has emerged. It has been characterized by a very high degree of ignorance and a very strong effect of surprise that has led to total confinement in almost every country in the world.

In response to this pandemic, to complete the academic year, several educational institutions around the world, including IMSIU, transferred, abruptly, in a few days, from traditional learning to EL. For several reasons, these institutions are the first that need to evaluate the effectiveness of their ELS as a result of their experience during the COVID-19 pandemic. First, the adoption of ELS, without being preceded by good planning under the effect of surprise, and the sudden change, can lead to significant costs, use of unattractive EL products and, consequently, to failure. Second, knowing the output of this experience is necessary to judge whether it succeeds or fails. To what degree? What are the reasons for success or failure? Third, it is necessary to know if they will continue with EL if COVID-19 pandemic persists in the following academic year (2021/2022). Fourth, it is necessary to plan the adoption of ELS in order to implement it in the future.

From this perspective, this research aimed to complement previous research by providing an e-learning effectiveness model through the evaluation of e-learning effectiveness (ELE) under the conditions of the pandemic of COVID-19, considering the main antecedents of ELE. Thus, this research aimed to achieve four objectives. Firstly, by developing an integrated model for the factors affecting ELE. Secondly, by identifying the sub-dimensions for the key antecedents of ELE in our research model. Thirdly, by assessing the scale validations for the key antecedents of ELE. Finally, by investigating the direct and indirect relationships among the key factors that influence ELE.

## 2. Theoretical Background

### 2.1. E-Learning Effectiveness

The increase in the adoption of EL in HEI has been followed by higher failure rates of many ELS [13]. This has led researchers to investigate the success and failure of EL [11,13,14]. Some reasons for failure are content, comfort level with technology, as well as availability of technical support [15], gaps in terms of three dimensions (ethical, evaluation, and management) [16], effective planning of ELS and a lack of experience [17]. On the other side, the key factors for the successful implementation of ELS are the presence of a culture favorable to EL among students [18], computer literacy, availability of appropriate technology, accessibility, and having a good high bandwidth internet connection [19,20].

The ELE concept is complex and multidimensional [1,2,12,21]. Despite this, many studies have attempted to examine the antecedents of ELE [22,23,24]; they did not introduce a comprehensive model for the antecedents of ELE. However, the results of these studies have not always been consistent [25,26] and, therefore, ELE antecedents remain unidentified. Thus, our research helped in filling this gap by introducing a comprehensive framework for the antecedents of ELE in higher education institutions during the COVID-19 pandemic in the KSA context. This framework has been adopted from different fields of research, such as management information systems, pedagogy, education, and psychology [27]. From this perspective, the systemic approach to EL could help to analyze and explain the effectiveness of EL as a dynamic set of interdependent sub-entities interacting together. Based on an inventory of a considerable number of models of ELSS, [4] suggests two dimensions to measure the effectiveness of ELS: net benefits (NB) and user satisfaction (US). NB involves the impacts of ELS, such as academic achievement, empowerment, learning enhancement, and time savings. US is the positive or negative responsiveness of a user toward the skills accumulated or knowledge enhancement through ELS.

### 2.2. The Key Antecedents of E-Learning Effectiveness

As such, to better understand the adoption of EL, it is important to examine the relevant factors influencing its effectiveness. The factors affecting the effectiveness of EL are several and various because researchers have regarded these factors in terms of student perceptions, pedagogical aspects, EL environment, technological support, societal factors. According to [28], these factors assimilated to challenges are course, characteristics of students or teachers, context (societal, cultural, and organizational), and technology. For other researchers, these factors were, namely, infrastructure, support of the system, e-learning readiness (ELR), learning culture, design system, resistance to change (RTC), and interactivity (INTRVAY) [21,29,30,31,32]. However, the problem with this research is related to the large number of variables that had a potential impact on ELE. To deal with this problem, we have focused on the variables most used by recent research (Table 1). Based on these 20 studies, it appears that effective ELS depends on the interactions with four variables: ELS, ELR, INTRVAY, and RTC.

#### 2.2.1. E-Learning System

Over the past two decades, it is evident that ICT has reshaped social life [46]. This evolution has affected the context of traditional education, which has undergone profound changes with the emergence of EL as an extension of traditional learning (face to face). It has been adopted by various HEI as an important part of learning. Because ICT is a main component of EL, it had several nominations, such as EL, network learning, distance learning, e-teaching, online learning, technology-mediated learning, virtual learning, distance education, web-based learning and distributed learning [47]; therefore, no generally accepted definition is available [48,49]. Previous scholars have defined EL as the use of a variety of electronic media to deliver learning to learners, including the interactive TV, intranet, satellite broadcast, extranet, and internet [1,48,49]. However, these visions are very reductive, since they limit the scope of the concept to a single technological component, while forgetting other basic components.

According to [50], EL is an outcome of combining theories of permanent learning and adult education. It will depend basically on adult learning theory and online collaborative learning theory to develop and build a comprehensive model for effective ELS in HEI. Regarding adult learning theory, [51] mentioned that “*it is focused mainly on how adults learn*”. He added that learning theory is built on five dimensions: motivation to learn, adult learner experience, orientation of learning, self-concept, and readiness to learn. Online collaborative learning (OCL) “*focuses on the facilities of the Internet to provide learning environments that foster collaboration and knowledge building*” [52]. Reference [53] defined OCL as: “*a new theory of learning that focuses on collaborative learning, knowledge building, and internet use as a means to reshape formal, non-formal, and informal education for the knowledge age.*” According to [54], EL builds mainly on the interaction between learning theories and ICT. It is networked, delivered via a computer using internet technology and goes beyond the classical paradigms of learning. It brings together critical thinking, organizational and analytical skills, oral and written communication, problem-solving skills, initiative-taking, and interaction with classmates and instructors. In a similar statement delivered by [55], EL was defined as “*the use of electronic media and information and communication technologies in education*”. From these definitions, EL goes beyond the simple use of a technological tool and goes beyond being the “*Use of internet technology for the creation, management, making available, security, selection and use of educational content to store information about those who learn and to monitor those who learn, and to make communication and cooperation possible*” [56].

Educational leaders in the United States, such as [57], explain that, if universities do not change radically from traditional learning to EL, they will cease to exist in the twenty-first century. That is why, in recent years, the integration of EL at the university around the world has become necessary, even urgent. As a strategic choice, [58] explains that external and internal factors encourage the development and implementation of EL in institutions’ academics. Indeed, the introduction of EL leads to the emergence of strategic challenges. Reference [25] lists ten challenges: hierarchical, organizational, managerial, legal, technical expertise, psychological, staff development, role of teachers, administrative and technical staff, student support, and funding. These different challenges assume that the implementation of EL at the university requires, a priori, a well-established planning phase.

Based on extant literature synthesis and validation, [4] carried out an extensive inventory of models of ELSS and came up with three dimensions of ELS that are validated empirically: service quality (SvQ), system quality (SyQ), information quality (IQ). SvQ involves instructor-student interactions on attributes such as promptness, availability, competency, fairness, and responsiveness. SyQ focuses on the characteristics of the website or an EL portal such as responsiveness, stability, user-friendliness, security, and ease of use. IQ is defined as the quality of content on aspects such as organization, presentation, length, and clarity.

#### 2.2.2. E-Learning Readiness

A considerable number of studies have considered ELR as a critical factor for the success of ELS [30,34,35]. The implementation of ELS can be preceded by measuring the level of ELR that allows institutions to shape a system adapted to the expected results in order to be a successful implementation. In fact, success in ELS depends on three pillars: the efforts of the educational institutions, those of the instructors, and student background. Understanding the components of this concept and discussing the theory of ELR makes it much easier to understand its impact on the success of ELS.

ELR is broadly defined as “The preparedness of the students to fully explore and exploit the learning opportunities provided by ICT and its related learning technologies, and, ultimately, to maximally draw the attendant benefits in terms of students’ academic achievements, reduced dropout rates, social connectivism and for lifelong learning” [59]. Simply, ELR is “those factors that must be accomplished before EL implementation can be regarded as being successful” [60]. This preparedness encompasses two main components, namely, computer technology and the process that learners go through. According to previous research related to ELR, these two components can be broken down into several aspects: students behavior, and student attitudes [61]; policy, technology, financial, human resources, infrastructures [62]; skills, and attitudes [63]; learner characteristics, behavioral engagement technology capabilities, student behavior, emotional engagement, student self-direction, cognitive engagement, student attitude [64]; skill, attitude, experience, organizational barrier, motivation [65].

These multiple aspects are integrated in a synthetic way by [66] by defining ELR under the following aspects: online communication self-efficacy (OCS), computer and internet self-efficacy (CIS), learner control (LC), motivation for learning (MFL), and self-directed learning (SDL). SDL means that the student is able to learn independently. He is able to take the initiative to understand his learning needs, set his learning objectives, identify and allocate his resources for learning, as well as to choose and apply appropriate learning strategies, and evaluate his learning results. MFL refers to the students’ enthusiasm and self-drive to participate in EL. It directs students’ efforts towards their own desires and to improve their learning, recovery, and retention. LC is related to the ability of the student to know how to learn as he makes learning decisions and considers experimental results of those decisions. CIS is related to the availability of computer devices and internet access and the ability of students to use them. Since EL is delivered via networks, it is necessary that all assessments be linked. OCS involves computer-mediated communication between students and between them and instructors.

#### 2.2.3. Interactivity

According to [29], interactivity (INTRVAY) is defined as “open operational interventions planning, problematized, that pushes the learner to find solutions based on what the previous knowledge suggests”. In traditional learning, INTRVAY is a fundamental tool to acquire knowledge [67], the heart of learning systems [68,69], and a natural attribute of face-to-face conversation [70]. In the context of ELS, INTRVAY, according to many studies, plays a vital role and has been gaining increasing interest as one of the buzzwords of the discussion on learning systems [71]. This evolution is due to the fundamental changes related to the move from teacher–student dependence design to a teacher–student independence design. In this independent design, INTRVAY takes four shapes: student–system, student–instructor, student–student, and student–content [69].

Student–content (StC) (cognitive presence) refers to how interactively the student can access the knowledge presented and accommodate it into his existing cognitive structure. Student–instructor (StI) (teaching presence) refers to how interactively the instructor delivers the content and the skills required for the student to access the content, by presenting, clarifying information, supporting, encouraging, evaluating, and providing feedback. student–student (StSt) (social presence) is related to the extent through which the students interact with their peers in order to exchange knowledge through communication. Student–system (StSy) refers to interactions not only between students and the interface of EL, but also between students and the other components of the system, which contains all the information that the users are trying the access. Having technological infrastructure alone is not enough to make EL successful [72]. Students need to control the interface tools to access the content, such as buttons, hyperlinks, and menus. Further, they need to interact with the other participants of the system, i.e., the teacher and other students, in many forms, such as quizzes, forums, online chats, video-conferencing and emails.

These four types of INTRVAY have been proven to have a positive effect on EL at several levels: effective student learning [73], increase in the level and speed of student learning [74], increase in learning enjoyment level [75], improvement of student motivation and confidence [76], improvement of student control, and persistence [46], strong student, satisfaction by student–content, and student–system interactions [77], engagement, communication, conversation, and control [78], strong student attention [79], generation of more information, more effective learning process, and more motivated students [80], concentration of ELS on the students [81]. On the other side, in contrast to the above advantages, INTRVAY in EL has some disadvantages: absence of vital personal interactions, not only between students and instructors but also among student colleagues [82]. Thus, from this previous review of the empirical literature, and for a better understanding of EL adoption, it is important to investigate the influence of INTRVAY in EL on ELE.

#### 2.2.4. Resistance to Change

One of the foundational challenges for the implementation of ELS to be a success is RTC [83,84,85]. It presents a challenge, as the brand-new system is still changing routines and behaviors, which are not accepted by a number of users (teachers, students, and administrators), thereby, producing RTC [86]. Previous ICT studies have shown that RTC is strongly related to the acceptance and use of new ICT [87]. This challenge becomes more serious when the change is surprising without any prior planning, such as the case of the COVID-19 pandemic [85].

Researchers identify several organizational problems of integration, use, development of ICT in education [83,84,86], work practices, underestimation, lack of follow-up, lack of awareness, user dissatisfaction with new systems, negative attitudes towards ICT, culture, lack of systemic approach to implementation, mismatches between technologies and the context, high rates of system non-completion, technical end-user support, lack of user training, and lack of administration. Other researchers explain RTC by six reasons related to the individual’s personality [88], such asthat individuals do not want to lose control of their own situation, which creates uncertainty and resistance in them (reluctance to lose control); dogmatic and fairly closed-minded individuals are reluctant to adapt to new situations (cognitive rigidity); efforts to change create a higher level of stress, and personal resilience reduces the ability to cope with these changes (lack of psychological resilience); individuals might resist change due to the increased workload and stress during the change (intolerance of the adjustment period involved in the change); adaptive individuals like familiarity and stick to the current framework, while innovative individuals look for new ideas and want to get out of the current framework (preferences for low levels of stimulation and novelty); individuals in a new situation may feel new stimuli and their normal response may be inappropriate in the new situation. This could lead to stress due to the misfit (reluctance to give up old habits).

## 3. Conceptual Model and Research Hypotheses

**Figure 1:**
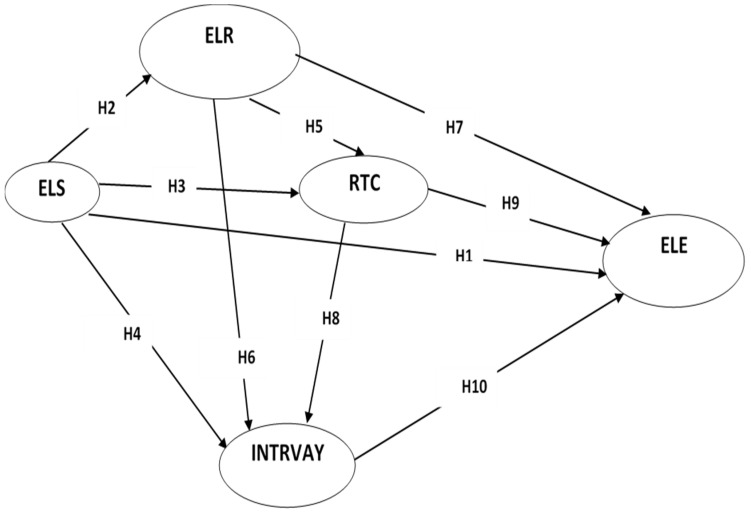
Conceptual model. ELS: E-learning System; ELR: E-learning Readiness; INTRVAY: Interactivity; RTC: Resistance to Change; ELE: E-learning Readiness.

As above, and with reference to the aforementioned literature review, in order to apprehend the reality of the developing ELE in HEI, precisely in the context of the COVID-19 pandemic, a conceptual model was proposed. This model focused on the assessment of ELE by considering four factors affecting ELE in terms of direct and indirect relationships: ELS [4,26,32,34,37,39], ELR [30,34,35], INTRVAY, [38,42,46,73,75,78,80,81] and RTC [32,34,45,88]. Integrating those factors resulted in the model depicted in Figure 1.

As presented in this proposed model, ten hypotheses (H1 to H10) were suggested, representing direct relationships between factors that affect ELE. On the other hand, six hypotheses (H11 to H16) were proposed, examining the indirect relationships (Amos program shows these relationships in the results) between these factors and with ELE. Therefore, the following hypotheses were suggested:

**Hypothesis** **1** **(H1).** *E-learning system affects e-learning effectiveness.*

**Hypothesis** **2** **(H2).** *E-learning system affects e-learning readiness.*

**Hypothesis** **3** **(H3).** *E-learning system affects resistance to change.*

**Hypothesis** **4** **(H4).** *E-learning system affects interactivity.*

**Hypothesis** **5** **(H5).** *E-learning readiness affects resistance to change.*

**Hypothesis** **6** **(H6).** *E-learning readiness affects interactivity.*

**Hypothesis** **7** **(H7).** *E-learning readiness affects e-learning effectiveness.*

**Hypothesis** **8** **(H8).** *Resistance to change affects interactivity.*

**Hypothesis** **9** **(H9).** *Resistance to change affects e-learning effectiveness.*

**Hypothesis** **10** **(H10).** *Interactivity affects e-learning effectiveness.*

**Hypothesis** **11** **(H11).** *E-learning system affects indirectly resistance to change via the mediating roles of e-learning readiness & interactivity.*

**Hypothesis** **12** **(H12).** *E-learning system affects indirectly interactivity through the mediating role of resistance to change.*

**Hypothesis** **13** **(H13).** *E-learning system affects indirectly e-learning effectiveness via the mediating roles of e-learning readiness, resistance to change & interactivity.*

**Hypothesis** **14** **(H14).** *E-learning readiness affects indirectly interactivity through the mediating role of resistance to change.*

**Hypothesis** **15** **(H15).** *E-learning readiness affects indirectly e-learning effectiveness via the mediating roles of resistance to change & interactivity.*

**Hypothesis** **16** **(H16).** *Resistance to change affects indirectly e-learning effectiveness via the mediating role interactivity.*

## 4. Research Methods

### 4.1. Data Collection

This research adopted an online questionnaire to collect our research data. Respondents were students from the three biggest colleges in IMISU according to number of students (College of Economics and Administrative Sciences, Fundamentals of Religion College, and Social Science College) for the second term of the academic year 2019–2020. Our criteria in selecting research samples from IMISU as the research population were: Firstly, these three colleges represented 53% of the total number of IMISU students. Those three colleges (research sample) adopted some online activities in their learning process before the COVID-19 pandemic (e.g., blackboard), which meant that their students as survey respondents had enough experience to assess the antecedents of ELE. Secondly, our sample represented the population in an effective way, where the sample represented the population in terms of gender and academic year.

In the same line of most studies, considering ELE, we focused on students’ perceptions. Firstly, students are important since they represent key users of ELS; all the actors in the university work at their service. Secondly, students are the end-users of ELS, who, when satisfied, will recommend the service to other students, and, consequently, the relationship with the service provider will continue [89,90]. Thirdly, students had continuous interactions with the ELS during the COVID-19 pandemic. Their opinions provide in-depth help in constituting a complete picture of ELE. The questionnaire was tested with 3 academicians, 3 researchers, and 30 students to enhance its content and clarity. After required modifications, the last form of the questionnaire was directed to 1202 students (Table 2).

### 4.2. Research Instrument Development-Measures

For all the variables of the research, the items of the questionnaire were developed from previous studies to ensure scale validation to our research constructs and their reliabilities. The scales’ development “*is based on the survey of extant theoretical items and a review of the literature*” [91]. Our research questionnaire engendered five main variables with their precise ingredients: ELS, ELR, RTC, INTRVAY, and ELE, which were altered for the research context. All the variables were measured through a five-point Likert scale that extended from strongly disagree (1) to strongly agree (5). ELS was measured through 18 items composed of SyQ, IQ &SvQ and adopted from [13,91,92,93,94,95]. Regarding ELR dimensions, they were measured through 18 items derived from [66]. The INTRVAY construct had been measured via 22 items from [96,97]. RTC construct had been measured through three items from [98]. Finally, ELE was measured via two dimensions: US (3 items) and NB (3 items), which were adopted from [4,99]. Following [100], “*translation and back translation procedure, the Arabian version from the original English scales was used for this research sample* “. For illustration, SyQ was measured by these items: the e-learning system provides high availability; the e-learning system is easy to use; the e-learning system is user friendly; the e-learning system provides interactive features between users and system; the e-learning system provides personalized information presentation; the e-learning system provides charming features to attract users; the e-learning system provides high-speed access of information.

An independent t-test was run; the results showed non-significant differences at 95% amid the respondents from the three colleges in IMISU, as recommended by [101]. Our t-test findings revealed that no significant differences existed, denoting that sample bias was not symptomatically problematic [102]. The sample bias was also assessed “*in terms of the difference between demographic characteristics against attitudinal variables by using tests such as the t-test, the one-way analysis of variance (ANOVA), and Tukey’s multicomparison post-hoc tests*” [102]. To examine the common variance problem, Harmon’s test (one-factor test) was used as recommended by [103]. The results illustrated that no sole variable was responsible for the majority of covariance, verifying that the issue of common variance is not completely accountable for our findings.

## 5. Research Results

### 5.1. Confirmatory Factor Analysis

This research used a confirmatory factor analysis (CFA) “*to evaluate overall model fit with the data and measure the unidimensionality of research constructs*” [91]. For assessing the CFA goodness of our research model, [104] indorsed that (x2/df) had to be fewer than 3; TLI (Tucker–Lewis index), CFI (comparative fit index), and NFI (Normed fit index) must surpass 0.9. On the other hand, RMSEA (root mean square error of approximation) should be ≤0.05. A joint confirmatory factor analysis, with all of the variables, was conducted using Analysis of a Moment Structures (AMOS) v20.0 [91]. Findings illustrated in Table 3 displayed the overall fit statistics. All CFA indices were acceptable, as all attained fit indices showed the suggested cut-off values. Moreover, Table 3 illustrates the results of the convergent validity for ELS, ELR, RTC, INTRVAY, and ELE that constitute our research constructs.

Findings verified the convergent validity as endorsed by [105], where all factor loading for all research construct dimensions were above 0.5 and were significant. Secondly, the average variance extracted (AVE) must be superior to 0.5. Finally, the construct reliability for all research constructs was bigger than 0.7. Regarding the discriminant validity assessment of constructs, as shown in Table 4, all AVEs were higher than the off-diagonal values; all alpha values were extended from 0.88 to 0.95, which were greater than the supreme value of correlation amongst any two couples of our research variables (0.77), as suggested by [105].

### 5.2. Structural Equations Modeling Results

**Figure 2:**
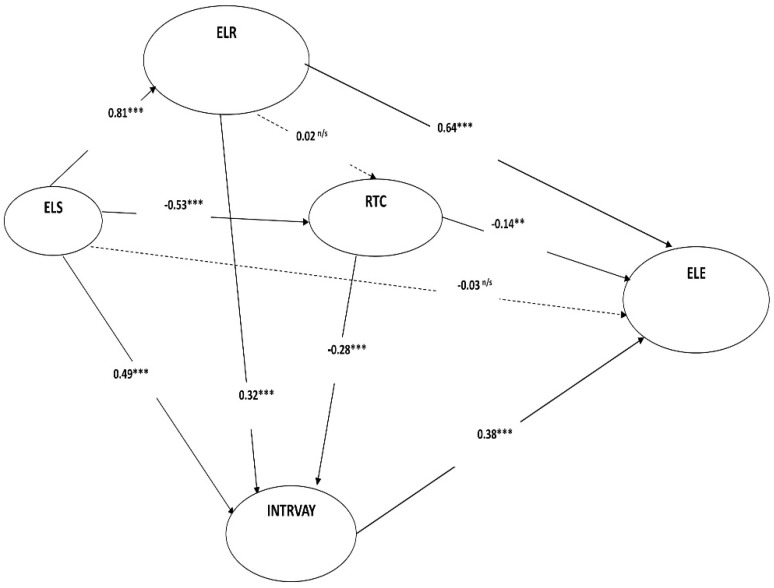
SEM results. ** *p* < 0.01; *** *p* < 0.00, n/s = not significant. ELS: E-learning System; ELR: E-learning Readiness; INTRVAY: Interactivity; RTC: Resistance to Change; ELE: E-learning Readiness.

Our hypotheses were examined by using structural equations modeling (SEM). The valuation of the anticipated model has been done using the following measures, which reflect the overall model goodness and the significance of our research hypotheses, as recommended by [102,107]. Our research model examined ten direct effect relationships and six indirect effect associations between our research constructs. Figure 2 and Table 5 illustrate the outcomes of direct effects among the proposed hypotheses. SEM results revealed that ELS had no significant effect on ELE (*β1 = −0.03 with p > 0.05*), which proved no support for *H1*.

Our results proved a strong direct positive and significant effect of ELS on ELR (*β2 = +0.81 with p < 0.01*), as suggested in *H2*. Our SEM findings showed that ELS had a negative and significant impact on RTC (*β3 = −0.53 with p < 0.01*), as proposed in *H3*. Similarly, ELS had a positive and significant impact on INTRVAY (*β4 = +0.49 with p < 0.01*), as expected in *H4*.

Concerning the results of direct relationships between research constructs, our findings showed a non-significant effect of ELR on RTC (*β5 = +0.02 with p > 0.05*) which did not confirm *H5*. On the other hand, ELR showed a positive and significant effect on INTRVAY (*β6 = +0.32 with p < 0.01*) and a strong positive and significant effect on ELE (*β7 = +0.64 with p < 0.01*), which verified *H6* and *H7*. Our findings also demonstrated that RTC had a negative and significant impact on INTRVAY and ELR (*β8 = −0.28 with p < 0.01; β9 = −0.14 with p < 0.05*), as proposed in *H8* and *H9*. Finally, findings showed a significant and positive impact of INTRVAY on ELE (*β10 = +0.38 with p > 0.01*), as recommended by *H10*.

Moreover, our research model investigated six indirect relationships among our research constructs, as recommended in *H11, H12, H13, H14, H15 & H16* (Table 6). The positive indirect effect of ELS on RTC via ELR and INTRVAY showed a weak indirect effect (*β11 for indirect impact* via *ELR & INTRVAY = +0.02*) that reduced the negative effect between them from −0.53 to −0.51, which showed that ELR & INTRVAY did not play an effective intermediating role in enhancing the linkage between ELS and RTC, as suggested in *H11*. As hypothesized in *H12*, our findings found that ELS had a positive indirect influence on INTRVAY via the intermediating role of RTC (*β12 for indirect impact* via *RTC = +0.41*) that raises the value of total effect by +0.41 (from +0.49 to +0.90). As we proposed in *H13*, ELS had a strong indirect effect on ELE via ELR, RTC and INTRVAY (*β13 for indirect impact* via *ELR, RTC & INTRVAY = +0.83*), which confirms *H13*. Findings also asserted that ELR had a weak indirect effect on INTRVAY in our model via RTC (*β14 for indirect impact* via *RTC = +0.01*), which increased the positive total effect between them from +0.32 to +0.33, thus, showing that RTC did not contribute strongly as a mediator in improving the effect of ELR on INTRVAY, as suggested in *H14*. As assumed in *H15*, findings showed that ELR had a positive effect on ELE via RTC and INTRVAY (*β15 for indirect impact* via *RTC & INTRVAY = +0.12*) that improved the positive total effect between them from +0.64 to +0.76, which showed that RTC and INTRVAY played important roles in improving the effect of ELR on ELE, as suggested in *H15*. Finally, RTC also had a negative effect on ELE via INTRVAY (*β16 for indirect impact* via *INTRVAY = −0.11*) that raised the value of total effect between them from −0.14 to −0.25 and showed that INTRVAY contributed strongly as a mediator in the effect of RTC on ELE, as suggested in *H16*.

## 6. Discussion

Assessing e-learning effectiveness in HEI, specifcally during crises such as the COVID-19 pandemic, is a questionable topic in e-learning literature. During this pandemic, most HEI around the world shut down and moved to e-learning because of the necessity for social distance to control and reduce the spread COVID-19. From this perspective, this research attempted to develop an integrative model for effective e-learning in HEI, such as colleges of IMSIU. This model included five constructs reflecting many interactions between e-learning readiness, interactivity, and resistance to change to promote e-learning effectiveness. Through the interactions between these variables, the three components (system quality, information quality, and service quality) of e-learning systems were validated, where information quality developed as the most significant contributor in establishing and developing e-learning system in IMSIU, with 0.94, followed by system quality, with 0.92, and, finally, ranked service quality, with 0.79. This classification invites any HEI to be very careful to the three components of the e-learning system, and, more precisely, information quality and system quality as recommended by prior research [2,109]. In the same vein, the five components (computer and internet self-efficacy, self-directed learning, learner control, motivation for learning, and online communication self-efficacy) of e-learning readiness were validated. In forming e-learning readiness, self-directed learning came first, with 0.93; learner control ordered second, with 0.87; online communication self-efficacy classified third, with 0.74; motivation for learning ranked fourth, with 0.71; computer and internet self-efficacy was the last, with 0.69. Thus, these five components, more precisely, self-directed learning and learner control, must be developed in order to be beneficial to e-learning effectiveness. The role of the HEI should encourage the student to control himself and to learn independently. This result contradicted some of the prior studies [64]. Regarding the scale validation of interactivity, findings presented that student–system comes first in creating interactivity, with 0.97; student–content ordered second, with 0.96; student–instructor classified third, with 0.86; finally, student–student came last, with 0.84. Thus, all components of interactivity were important to support e-learning effectiveness, which is compatible with the results of previous studies, such as [38,76]. Moreover, student–system and student–content had more importance and needed more attention. HEI needs to focus on developing a platform that is suitable and interactive, and that facilitates access to the knowledge presented in the platform. Finally, the scale validation to e-learning effectiveness constructs verified that user satisfaction had a powerful influence in developing e-learning effectiveness, with 0.95, whereas net benefits ranked second with 0.92. This result confirmed the research results of [4,26].

Consistent with our suggestions in direct relationships in our research model, e-learning systems contributed positively in reducing resistance to change, but need to integrate with interactivity. Thus, e-learning readiness, resistance to change, and interactivity play mediating roles between e-learning systems and e-learning effectiveness. For illustration, e-learning systems contribute significantly in increasing the level of interactivity between system users and the other elements of e-learning systems and in strengthening the readiness of students for studying online. These results justify why most e-learning system students pay more attention to information quality as one of the main dimensions in building a high-quality system. As hypothesized, our SEM results confirmed that increasing the level of e-learning readiness to adapt with e-learning systems through improving computer and internet self-efficacy, self-directed learning, learner control, motivation for learning, and online communication self-efficacy will enhance e-learning effectiveness. Moreover, a high level of e-learning readiness to study online enhanced the level of interactivity between e-learning system elements and had no effect on reducing the students’ resistance to change toward studying online. As expected in the relationship between resistance to change, interactivity, and e-learning effectiveness, our results showed inverse relationships between them, where a high level of resistance to change decreased the level of interactivity and minimized e-learning effectiveness.

According to indirect relationships in our research model, our findings verified that system quality enhanced e-learning effectiveness through obtainability of the high level of interactivity, high degree of e-learning readiness, and low level of resistance to change. E-learning readiness also increased e-learning effectiveness through availability of a high degree of interactivity and low level of resistance to change. Moreover, resistance to change negatively affected e-learning effectiveness through reducing the level of interactivity.

## 7. Conclusions

The synthesis of the previously discussed different results essentially led to the general conclusion that e-learning effectiveness depends on the interactions between four dimensions: the e-learning system, e-learning readiness, interactivity and resistance to change.

## 8. Theoretical and Managerial Implications

This research introduced some significant theoretical implications for both the knowledge and interactive fields of the e-learning literature. Firstly, our findings introduced an integrated model of e-learning effectiveness that represents the main aim of this research. This model can contribute significantly to building and establishing the online education theory suggested by [110]. Precisely, the research findings revealed that e-learning systems are not the only vital ingredient for effective online learning. Other vital ingredients, such as e-learning readiness and interactivity contribute significantly to enhancing the effect of e-learning systems on e-learning effectiveness. This relationship should not be considered by researchers as only a direct relationship, but rather as an indirect relationship through e-learning readiness, interactivity, and resistance to change. Therefore, successful and effective e-learning depends on the interactions between e-learning systems, e-learning readiness, interactivity, and resistance to change. Secondly, this research statistically validated comprehensive, multi-dimensional measures of the key antecedents for e-learning effectiveness, which include: e-learning system, e-learning readiness, interactivity, and resistance to change in HEI. Thirdly, our results verified that four key factors contribute to promoting an effective e-learning system.

Relating to the managerial implications of this research, HEI, especially in KSA, will be able to rely on our research findings to develop an effective e-learning system, especially in emergency situations and during long-term crises such as the COVID-19 pandemic, or even in normal situations by using hybrid education learning systems. HEI should know that the best strategy to enhance the effectiveness of e-learning is through increasing the quality of e-learning systems via continuous improvement in information quality, system quality, and service quality, where growing the quality of e-learning systems will increase student readiness to be comfortable with using e-learning systems and will increase their interactivity with all elements of the learning process, such as peers, instructors, online course content, e-learning tools, and student–support system. Moreover, high-quality e-learning systems will reduce resistance to change toward using e-learning systems. Finally, this research highlighted that, for an e-learning system to be effective, it must be a dynamic process that integrates multiple direct and indirect associations amid four constructs: e-learning system, e-learning readiness, resistance to change, and interactivity.

## 9. Limitations and Future Research

This research has two limitations that it is necessary to underline. Firstly, it was essentially based on data collection using a cross-sectional method. For more in-depth understanding to examine the “cause-effect” among our model constructs across time, we recommend adopting longitudinal research that gives more robust causality to the interactions between the key antecedents for an e-learning effectiveness model. Secondly, this research was conducted across the three biggest colleges in IMISU (College of Economics and Administrative Sciences, Fundamentals of Religion College, and Social Science College), which cannot generalize the findings to all colleges in IMISU or to all universities in Saudi Arabia. Therefore, our e-learning effectiveness model remained limited only to the three biggest colleges of IMISU. Despite these two limitations, our research introduced some promising avenues of research. Firstly, our e-learning effectiveness model was examined within an intra-university logic, emphasizing only three faculties. It is recommended to conduct further research in an inter-university logic by comparing several universities in Saudi Arabia. Secondly, researchers can investigate the role of control variables such as age, gender, department, and academic year in examining the impact of the e-learning system, e-learning readiness, interactivity, and resistance to change on our e-learning effectiveness model. These control variables could influence the findings of the research model by adding value to e-learning theory.

Thirdly, researchers can test our e-learning effectiveness model in other types of educational institutions, such as primary school, preparatory school, and secondary school, to recognize the antecedents of e-learning effectiveness in these institutions. Fourthly, researchers can further develop our e-learning effectiveness model by adding new relationships between e-learning systems, and e-learning effectiveness via e-learning readiness, interactivity, and resistance to change. For example, examining the direct effect of e-learning readiness, resistance to change, and interactivity on e-learning systems could add value to the establishment of a comprehensive model in e-learning effectiveness. Finally, upcoming studies are needed to investigate if the suggested relationships in our e-learning effectiveness model are valid in diverse cultural contexts. In fact, in the literature that is interested in studying the impact of cross-cultural on ITC, researchers have shown differences among people in their acceptance and preference toward using patterns of ITC [111]. Further research could pay more attention to comparative studies between divergent cultures to identify the impact of these cultural aspects and differences in our e-learning effectiveness model. Finally, further studies can examine our ELE model in different KSA universities and in different universities in other countries, especially in eastern cultures.

## Figures and Tables

**Table 1:** Antecedents of ELE.

Source	ELS	ELR	INTRVAY	RTC	ELE
[33]	*				*
[23]					*
[34]		*			*
[26]	*				*
[35]		*			*
[22]					*
[36]					*
[26]					*
[32]	*			*	
[37]	*				
[38]			*		*
[4]	*				*
[39]	*				*
[40]	*				
[41]	*				*
[42]	*		*		*
[43]	*			*	
[44]	*				*
[12]	*				*
[45]				*	*

*: Antecedents of E-learning studied; ELS: E-learning System; ELR: E-learning Readiness; INTRVAY: Interactivity; RTC: Resistance to Change; ELE: E-learning Readiness.

**Table 2:** Respondents’ characteristics (*N* = 1202).

Demographic Features	Variables	Usable Cases	%
Gender	Male	505	42%
	Female	697	48%
Academic year	Year 1	276	23%
	Year 2	240	20%
	Year 3	433	36%
	Year 4	253	21%
College	College of Economics and Administrative Sciences	480	26%
	Social Science College	409	34%
	Fundamentals of Religion College	313	40%

https://imamu.edu.sa/en/about/Pages/statistics.aspx (accessed on 2 February 2022).

**Table 3:** The assessment of convergent validity.

Construct	Factor Loadings	AVEs	Construct Reliability
ELS:		0.79	0.92
SvQ	0.79		
IQ	0.94		
SyQ	0.92		
ELR:		0.63	0.89
CIS	0.69		
SDL	0.93		
LC	0.87		
MFL	0.71		
OCS	0.74		
RTC:		0.73	0.89
RTC1	0.72		
RTC2	0.91		
RTC3	0.91		
INTRVAY:		0.83	0.95
StSy	0.97		
StC	0.96		
StI	0.86		
StSt	0.84		
ELE:		0.87	0.93
US	0.95		
NB	0.92		

Note: Goodness-of-Fit Indices: x2/df = 2.8, GFI = 0.90, CFI = 0.95, TLI = 0.94, NFI = 0.95, RMSEA = 0.039; Cut-off values for: Factor loading ≥ 0.5, AVE ≥ 0.5, Construct reliability ≥ 0.7. All standardized loadings are significant at the 0.01 level or better. x2/df =Chi-square/degree of freedom, CFI = comparative fit index, TLI = Tucker–Lewis index, RMSEA = root mean square error of approximation, RMR = root mean residual.

**Table 4:** The assessment of discriminant validity.

Variables	α	ELS	ELR	RTC	INTRVAY	ELE
ELS	0.92	0.89				
ELR	0.89	0.71 **	0.79			
RTC	0.88	−0.52 **	−0.41 **	0.85		
INTRVAY	0.95	0.76 **	0.74 **	−0.54 **	0.91	
ELE	0.95	0.77 **	0.73 **	−0.61 **	0.75 **	0.93

Note 1: ELS: E-learning System; ELR: E-learning Readiness; INTRVAY: Interactivity; RTC: Resistance to Change; ELE: E-learning Readiness.Note 2: ** “Correlation is significant at the 0.01 level (2-tailed), α = Composite Cronbach Alpha” [106]. Note 3: “Diagonal elements (in bold) are the square root of the average variance extracted (AVE). Off-diagonal elements are the correlations among constructs. For discriminant validity, diagonal elements should be larger than off-diagonal elements” [102].

**Table 5:** SEM results for the suggested model.

Predictor Variables	Criterion Variables	Hypothesized Relationship	Standardized Coefficient
ELS	ELE	H1→Not Support	−0.03 ^n/s^
	ELR	H2→Support	0.81 ***
	RTC	H3→Support	−0.53 ***
	INTRVAY	H4→ Support	0.49 ***
ELR	RTC	H5→Not Support	0.02 ^n/s^
	INTRVAY	H6→ Support	0.32 ***
	ELE	H7→ Support	0.64 ***
RTC	INTRVAY	H8→ Support	−0.28 ***
	ELE	H9→ Support	−0.14 **
INTRVAY	ELE	H10→ Support	0.38 ***
The obtained indices: x2/df = 2.72, GFI = 0.98, CFI = 0.99, TLI = 0.99, NFI = 0.99, RMSEA = 0.038.			
The cut-of values for goodness-of-fit indices: x2/df ≤ 3, GFI, CFI, TLI, NFI ≥ 0.9, and dd RMSEA < 0.05			

** *p* < 0.01; *** *p* < 0.00, n/s = not significant [91].

**Table 6:** Direct, indirect, and total effects among research variables.

Criterion Variable	Predictor Variables	Direct Effect	Indirect Effect *	Total Effect **
RTC	ELS	0.53	0.02	−0.51
INTRVAY		0.49	0.41	0.90
ELE		−0.03	0.83	0.80
INTRVAY	ELR	0.32	0.01	0.33
ELE		0.64	0.12	0.76
ELE	RTC	−0.14	−0.11	−0.25

ELS: E-learning System; ELR: E-learning Readiness; INTRVAY: Interactivity; RTC: Resistance to Change; ELE: E-learning Readiness. * “Indirect effects were computed only for cases in which the relevant structural parameters were statistically significant” [108]. ** “Insignificant direct effects were not included in the computation of total effect” [108].

## Data Availability

Data is available upon request from researchers who meet the eligibility criteria. Kindly contact the first author privately through e-mail.
